# Antibody-Based Imaging of Lymphatic Architecture in Murine Kidneys

**DOI:** 10.34067/KID.0000000919

**Published:** 2025-08-08

**Authors:** Arin L. Melkonian, Gelare Ghajar-Rahimi, Malgorzata M. Kamocka, Tarek M. El-Achkar, Timmy C. Lee, James F. George, Anupam Agarwal

**Affiliations:** 1 Division of Nephrology, Department of Medicine, University of Alabama at Birmingham, Birmingham, Alabama; 2 Department of Biomedical Engineering, University of Alabama at Birmingham, Birmingham, Alabama; 3 Division of Nephrology, Department of Medicine, Indiana University School of Medicine, Indianapolis, Indiana; 4 Indianapolis Veterans Affairs Medical Center, Indianapolis, Indiana; 5 UAB Veterans Affairs Medical Center, Birmingham, Alabama; 6 Division of Cardiothoracic Surgery, Department of Surgery, University of Alabama at Birmingham, Birmingham, Alabama

**Keywords:** AKI, ischemia-reperfusion, kidney, renal injury, renal ischemia, imaging

## Abstract

**Key Points:**

- Optimization of an antibody-based method for imaging murine kidney lymphatics.
- Accessible approach to studying difficult to visualize lymphatic vessels.
- Novel tools that can be used to investigate kidney-specific disease states.

**Background:**

The lymphatic system has historically been understudied due to the difficulty of distinguishing it from the blood vasculature. In AKI, the lymphatic system undergoes a process of expansion termed lymphangiogenesis. Current mainstay treatments inadequately treat the clinical consequences of AKI. Understanding kidney endothelial architecture and modifying its functional properties can serve as a promising therapeutic strategy for ameliorating AKI of multiple etiologies. A current barrier in lymphatic-modulating therapies is our inability to comprehensively examine the structural and functional integrity of kidney lymphatics. Current methodology to detect lymphatics includes traditional sectioning and immunolabeling for lymphatic markers. Because of the sparsity of kidney lymphatics, there may be significant variability in observed vessel density when using traditional sectioning and immunolabeling.

**Methods:**

Here, we describe a methodology adapted from the immunolabeling-enabled three-dimensional imaging of solvent cleared organs tissue clearing pipeline for visualizing intact lymphatic architecture at the organ level, providing a comprehensive analysis of the kidney lymphatic system. More specifically, we describe a detailed protocol outlining key steps in kidney tissue preparation, immunolabeling with two canonical lymphatic markers (vascular endothelial growth factor receptor-3 and lymphatic vessel endothelial hyaluronan receptor 1), optical clearing, imaging, and quantifying important lymphatic features such as vessel diameter, branch points, total volume, total length, and average segment length to assess the extent of lymphangiogenesis in injured kidneys. We use the software program *Imaris* to analyze three-dimensional kidney images and quantify their lymphatic features.

**Results:**

We have optimized an antibody-based approach for visualizing kidney lymphatics using confocal microscopy, offering a new approach to study lymphatics in the kidney. Quantification of lymphatic features reveals that injured kidneys have increased numbers of branch points, total volume, and filament length suggesting ischemia-induced kidney injury results in lymphangiogenesis.

**Conclusions:**

We have optimized an antibody-based method for imaging historically hard-to-visualize adult murine kidney lymphatics and developed a user-friendly approach for quantifying key lymphatic features that define lymphangiogenesis.

## Introduction

The kidney is composed of many cell types arranged in a complex microarchitecture.^1^ For example, filtration of materials from the glomerular and tubular epithelial system and reabsorption by the peritubular capillaries depends largely on the spatial relationship of the nephron with its surrounding structures.

Fluid-structure interactions are well studied in the context of blood vasculature. It is understood that macroscopic anatomy has physiologic relevance. For instance, the angle and diameter of arteriovenous fistulas influence blood flow dynamics and fistula success rates.^2^ Structural organization of lymphatic vessel (LV) networks similarly influences their functional capacity. While clinical methodologies of visualizing and assessing functional lymphatic anatomy have made great strides in recent years using novel radiotracers and advanced imaging modalities,^3^ preclinical methods studying intraorgan lymphatics, beyond the integumentary system, have predominantly centered on fluorescence microscopy.

Most kidney lymphatic studies have relied on standard two-dimensional microscopy of immunolabeled tissue sections. Although these methods have laid the groundwork for our understanding of lymphatic biology, they must be interpreted with caution with several caveats in mind:

- In a healthy kidney, capillary lymphatics are exceedingly sparse and often collapsed on histologic sections, making identification challenging—any given section may show limited lymphatic staining, making it difficult to determine if positive staining represents a lymphatic endothelial cell (LEC) lining a vessel's lumen or a free-floating cell within the parenchyma. The inability to distinguish lymphatics is exacerbated when relying on expression of a single molecular marker, as prototypical lymphatic markers are heterogeneously expressed along kidney LVs.^4–7^
- Two-dimensional imaging precludes full appreciation of the greater lymphatic network within the kidney. Specifically, characteristics such as branching density and overall lymphatic volume remain unknown when only analyzing a two-dimensional snapshot. By contrast, three-dimensional (3D) imaging provides a more comprehensive spatial representation of lymphatic vasculature and improves assessment of qualitative and functional characteristics of this network.

**Figure 1:**
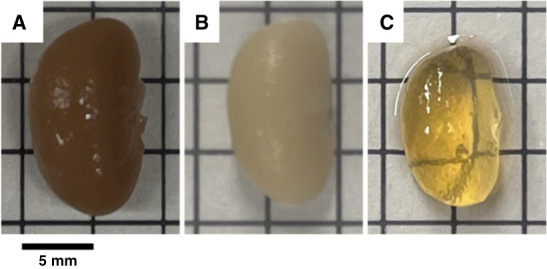
**Progressive change in kidney transparency.** Photographs of the intact murine kidney (A) immediately after fixation, (B) decoloration, and (C) RI matching in DBE. As seen, the tissue opacity is drastically reduced. Grid lines=5 mm. DBE, DiBenzyl Ether; RI, refractive index.

A significant limiting factor in imaging complex, dense organs is that light is scattered and absorbed by lipids, water, and proteins, which limits penetration depth in tissue.^8,9^ Reconstruction of 3D organ-level structures using standard light or fluorescence microscopy necessitates imaging copious numbers of serial sections.^10^ Altering tissue opacity, called “optical clearing,” offers a strategy to improve light penetration into tissue (Figure 1) and enable imaging of thick tissue sections or whole organs.^11,12^ The use of this principle has been documented as early as 1914 by Spalteholz^13,14^; however, only two groups have used such techniques to quantify murine kidney lymphatics to date.^15,16^

Jafree *et al.* used a modified immunolabeling-enabled 3D imaging of solvent cleared organs (iDISCO) clearing protocol along with antibody staining of two lymphatic markers, prospero homeobox protein 1 (Prox1) and LV endothelial hyaluronan receptor 1 (LYVE-1), to visualize LVs in embryonic murine kidneys and 1-mm thick sections of human fetal kidneys using confocal microscopy.^16^ Liu *et al.* used a modified clear, unobstructed brain/body imaging cocktails and computational analysis clearing protocol to visualize lymphatics in Prox1-tdTomato reporter mice using whole neonatal murine kidneys and 1-mm thick sections of adult murine kidneys with light-sheet microscopy.^15^ These studies quantified lymphatic vasculature using different analytical pipelines involving combinations of software and custom matrix laboratory and Fiji scripts. The use of reporter mice, arduous clearing protocols, light-sheet microscopy, and complex computational and image analysis create various bottlenecks in visualizing and quantifying kidney lymphatic vasculature at the organ level.

In this work, we have optimized the iDISCO clearing protocol for whole-mount microscopy and antibody-based visualization of adult murine kidney lymphatics. We propose the use of a pipeline for quantitative assessment of lymphatic architecture that is user-friendly and does not require advanced knowledge of image analysis. In addition, our approach is compatible with confocal microscopy and does not require the use of reporter mice, making the inclusion of lymphatics in scientific investigations more accessible.

## Methods

### Animals

C57BL6/J male mice (10 weeks) were used for this investigation. Nineteen minutes of bilateral ischemia-reperfusion injury (BIRI) and sham-surgical procedure were performed, as previously described.^17,18^ All animal procedures were performed in accordance with National Institutes of Health guidelines regarding the care and use of live animals and approved by the Institutional Animal Care and Use Committee of the University of Alabama at Birmingham (animal protocol number 21073, approved from 2023 to 2026). Quantification of LVs was performed on three sham and three BIRI mice to demonstrate reproducibility.

### Paraformaldehyde Preparation

PFA was prepared in batches and frozen in aliquots. Because PFA inactivation during liquid storage at 4°C affects tissue fixation and image quality, the same batch was used per experiment.^19,20^ To prepare 500 ml of 4% (wt/vol) PFA, 450 ml of water was heated to 55°C–58°C in a fume hood. Note, the solution should not exceed 60°C. In total, 20 grams of PFA (Sigma-Aldrich, 158127) were added, while the solution is stirred gently and 1 M sodium hydroxide (approximately 200 *µ*l) was added 1 drop at a time until the PFA was completely dissolved. 10× PBS was then added to adjust the volume to 500 ml and pH was monitored and adjusted to 7.4 as needed. The solution was cooled on ice, passed through a 0.45 *µ*m filter, aliquoted, and stored at −20°C. PFA stocks were thawed overnight at 4°C and passed through a 0.45 *µ*m nylon syringe filter immediately before use.

### Tissue Procurement

**Figure 2:**
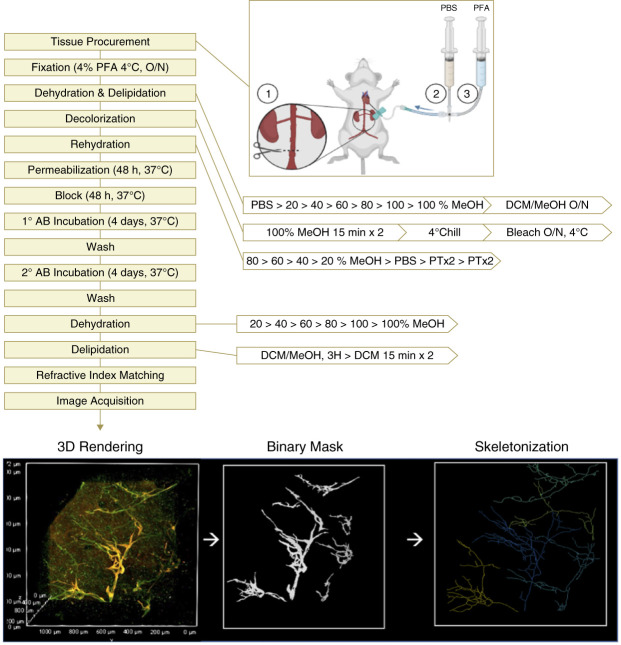
**Overall schematic of tissue collection, treatment, imaging, and computational workflow for visualization and quantification of murine kidney lymphatics using tissue clearing and whole-mount microscopy.** The dehydration protocol consists of sequential 1-hour washes, beginning with PBS followed by a gradient of increasing methanol concentrations. This series terminates in either a 66% DCM/33% methanol solution (delipidation) or 100% methanol. For rehydration, the process is reversed with 1-hour washes starting with 80% methanol and finishing on PTx2. AB, antibody; DCM, dichloromethane; MeOH, methanol; O/N, overnight; PFA, paraformaldehyde; PTx2, 0.2% Triton X-100 in PBS.

The general workflow of the following protocol is depicted in Figure 2. Ten-week-old mice were anesthetized with isoflurane, and kidneys were perfused. After exposing the abdomen, an incision was made in the abdominal aorta below the renal arteries. Ten mL of ice-cold PBS was pushed through a 21-gauge syringe inserted into the left ventricle, followed by perfusion with 10 ml of ice-cold, filtered 4% PFA which was freshly prepared from a frozen stock. PBS and PFA were passed through a 0.45 *µ*m nylon syringe filter before use to eliminate any particulates that may interfere with imaging. Uniform blanching of the lungs was used as a visual guide for perfusion efficiency. After perfusion, kidneys were excised and carefully decapsulated.

Tissues were submerged in ice-cold 4% PFA and kept at 4°C for at least 24, and up to 72, hours. After 4% PFA incubation, tissues were either selected for tissue clearing or transferred to 0.25% PFA for long-term storage at 4°C.

### Tissue Clearing and Immunolabeling

The iDISCO protocol^21^ was optimized for antibody-based visualization of kidney lymphatics in mice. Each kidney was cut into quarters by making one transverse cut and one sagittal cut using a razor blade. All steps were performed in 1.5 ml microcentrifuge tubes filled to the brim. Tissue was dehydrated with methanol at room temperature (RT) starting with a 1-hour incubation in 20% methanol in water, and the percentage of methanol was increased by 20% each hour until the tissues were in 100% methanol. Tissues were left in fresh 100% methanol for an additional hour and then chilled to 4°C. Tissue was incubated overnight in a delipidation solution (two parts dichloromethane and one part methanol) at RT while shaking. After 16 hours, tissues were washed (100% methanol for 30 minutes×2 at RT), chilled to 4°C, and then transferred to decolorization solution composed of freshly prepared and chilled 5% hydrogen peroxide in methanol. Tissues were incubated in decolorization solution overnight at 4°C. The following morning, tissues were rehydrated in a descending methanol series (80%, 60%, 40%, and 20% methanol in water, 1 hour each), followed by PBS (1 hour) at RT. Tissues were then washed in 0.2% Triton-X 100 in PBS (PTx2) buffer (1 hour×2) at RT.

After pretreatment, tissues were permeabilized for 48 hours in PTx2 with 20% DMSO, 2.3% (weight/volume) glycine at 37°C and then incubated in blocking solution (PTx2 with 6% donkey serum, 10% DMSO) for 48 hours at 37°C. The primary antibody was prepared in PBS containing 0.2% Tween-20 and 10 mg/L heparin (PTwH) with 5% DMSO and 3% donkey serum. Antibody stocks were spun at max speed for 5 minutes at 4°C before the antibody was removed from the top. Kidney quarters were incubated with two LEC markers, LYVE-1 (1:200 dilution; Novabus, nb-6008) and vascular endothelial growth factor receptor-3 (VEGFR-3; 1:200 dilution; R&D, AF743) for 96 hours at 37°C. Tissues were then washed in PTwH (1 hour×5) at RT and left over night in the fifth wash at RT. Secondary antibodies (1:300 dilution; AF594 LYVE-1 and AF647 VEGFR-3) were prepared in PTwH with 3% donkey serum, and kidney quarters were incubated for 96 hours at 37°C in the dark. Tissues were washed in PTwH (1 hour×5) at RT and left in the fifth wash for 16 hours at RT.

After immunolabeling, kidney quarters were dehydrated in methanol (20%, 40%, 60%, 80%, and 100% methanol in water, 1 hour each) and left in 100% methanol for 16 hours at RT. Tissues were then incubated in delipidation solution for 3 hours at RT on a shaker. Methanol was washed off with 100% dichloromethane (15 minutes×2 while shaking). Finally, tissue was placed in DiBenzyl Ether (DBE, Thermo, AC148400010; refractive index [RI]=1.424) to perform RI matching. It is crucial to select an appropriate immersion media that matches the RI of the tissue for optimal imaging. Cleared tissues can be stored in a microcentrifuge tube filled to the brim with DBE for up to to 12 months if shielded from light and stored in a cool place.

### Image Acquisition with Confocal Microscopy

Confocal images (1.24 *µ*m/px) were captured with a Nikon A1R Confocal microscope with the Nikon air 10× objective (Plan Apo *λ* 10×, numerical aperture. 0.45, wd 4000, RI 1.0) and sequential resonant scanner at the University of Alabama at Birmingham High-Resolution Imaging Facility. The emission and excitation wavelengths for AF594 and AF647 are 595/561.7 and 700/637.9 nm, respectively. Tissues were placed in the center of the glass coverslip of a disposable glass bottom dish (MatTek, P35G-0-7-C). A small drop of DBE was placed onto the tissue to prevent tissue shrinkage during image acquisition. The image stitching function of Nikon Imaging Software was used in combination with z-stacks with 1% overlap between tiles for image capture.

### Quantification of Lymphatic Vasculature in the Kidney

Quantifying features from 3D images requires image segmentation to set boundaries and define objects of interest, followed by creating binary masks for each fluorescence channel. Key parameters for microvasculature structures (*e.g*., length, diameter, volume, and branch points) are calculated using skeletonization and the Filaments feature. Before Imaris import, raw.nd2 files were first denoised and deconvolved using the Richardson-Lucy algorithm in the Nikon Imaging Software and then converted to .ims format for analysis.

### Imaris Pipeline (Version 10.2.0)

1. Image Optimization: Files were imported into Imaris and lookup table settings were individually optimized for each mouse to visualize lymphatics.
2. Surfaces: A surfaces module was created for each channel. Surface creation used whole-image analysis with mouse-specific absolute intensity thresholds to accommodate for background variation. Standardized parameters (“number of voxels Img=1” and “sphericity”) were applied across all mice. After execution of the Surfaces function, nonspecific, nonlymphatic background elements were manually eliminated using the pencil delete tool. This step is *critical* for ensuring that all features analyzed are lymphatic in nature. From the manually refined Surfaces image, a binary mask was generated.
3. Filament Analysis: A filaments module was created with only the “object-object statistics” option enabled. The channel-specific binary mask was selected as the working area, and analysis was performed using the “multicheck points” feature. Diameter parameters were defined as 1–30 *μ*m. The Detection Type excluded soma and spine. The algorithm was trained to accurately discriminate points and filament branches before execution, after which statistical data were extracted from the software using the toolbar.

This pipeline was executed sequentially, first analyzing the LYVE-1 channel followed by the VEGFR-3 channel. All machine learning parameters were initially optimized using an injured mouse image, and these standardized parameters were then applied uniformly across all experimental groups to minimize bias.

LYVE-1 surfaces: Number of voxels Img=1 above 173, sphericity between 0.0566 and 0.563. LYVE-1 filaments: Seed points threshold 273. VEGFR-3 surfaces: Number of Voxels Img=1 above 75, sphericity between 0.0519 and 0.665. VEGFR-3 filaments: Seed points threshold 120.

## Results

**Figure 3:**
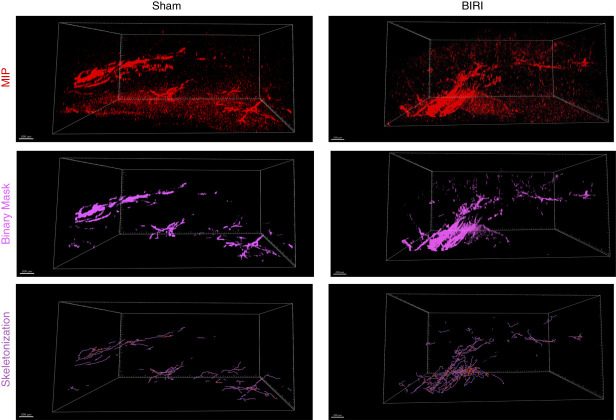
**Visualization of LYVE-1**^**+**^ **LVs in a quarter of a mouse kidney 10 days after sham or BIRI operation.** Images were acquired using a 10× objective and resonant scanner. Images were preprocessed with algorithms to denoise and deconvolute data before import into Imaris to generate 3D reconstructions. Top panel shows the MIPs, middle panel shows the binary mask of LYVE-1^+^ vessel structures generated using the Surfaces tool on *Imaris*, and the bottom panel shows filament segmentation. 3D, three-dimensional; BIRI, bilateral ischemia-reperfusion injury; LV, lymphatic vessel; LYVE-1, lymphatic vessel endothelial hyaluronan receptor 1; MIP, maximum intensity projection.

**Figure 4:**
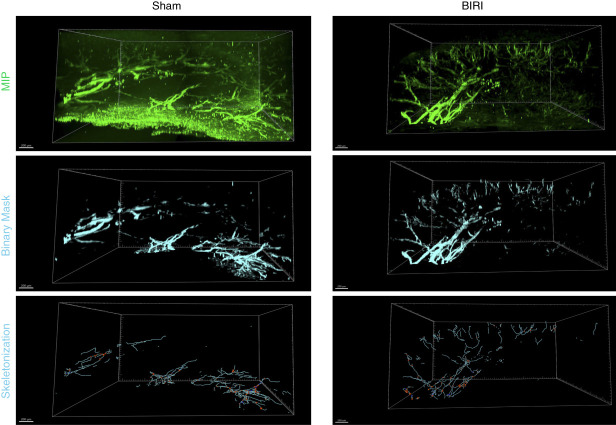
**Visualization of VEGFR-3**^**+**^ **LVs in a quarter of a mouse kidney 10 days after sham or BIRI operation.** Images were acquired using a 10× objective and resonant scanner. Image was preprocessed with algorithms to denoise and deconvolute data before import into *Imaris* to generate 3D reconstructions. Top panel shows the MIPs, middle panel shows the binary mask of VEGFR-3^+^ vessel structures generated using the surfaces tool on Imaris, and the bottom panel shows filament segmentation. VEGFR-3, vascular endothelial growth factor receptor-3.

**Figure 5:**
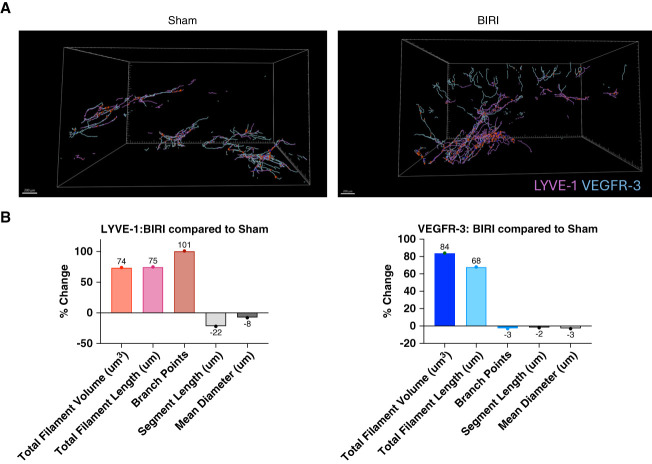
**Quantification of LYVE-1**^**+**^ **and VEGFR-3**^**+**^ **structures in sham versus BIRI kidneys.** (A) Simultaneous visualization of LYVE-1 and VEGFR-3 filament segmentation in sham and BIRI tissue, showing the 3D reconstruction of lymphatic networks. (B) Comprehensive quantitative analysis of lymphatic parameters showing percent changes for five key metrics: branch points, mean vessel diameter, total filament volume (sum of all filament volumes), total filament length (sum of all filament lengths), and average segment length. The bar graphs display the average percentage change in BIRI relative to sham controls (*n*=3 mice per treatment group). All raw values before percentage calculations were normalized to absolute volume obtained from imaging parameters.

We optimized an iDISCO tissue-clearing method to allow visualization of LVs in kidneys of adult mice using immunostaining and confocal microscopy. This approach enabled us to image at depths up to 1400 *µ*m thick by confocal microscopy. Reconstructed 3D renderings successfully visualized network architecture of LYVE-1 (Figure 3) and VEGFR-3 (Figure 4) positive LVs in detail. Costaining with both LYVE-1 and VEGFR-3 showed that while there was significant overlap of these two lymphatic markers, expression of these lymphatic markers was not entirely homogenous along the lymphatic vasculature (Figure 5A).

To determine whether structural alterations of lymphatic vasculature occur after AKI, we applied our imaging approach to murine kidney tissue collected 10 days after BIRI or sham surgical procedure. Quantification confirmed qualitative observations of expanded and altered LV architecture in injured kidneys compared with controls.

LYVE-1^+^ lymphatics exhibited increased total LV volume (sum; 74%), number of branch points (101%), and total filament length (sum; 75%), while decreased average segment length (−22%) and average mean diameter (−8%) relative to sham controls (Figure 5B). By contrast, VEGFR-3^+^ lymphatics showed an increased total LV volume (84%) and total filament length (68%; Figure 5B). For accurate representation of data, all raw values were divided and normalized by the absolute imaging volume (x, y, z depths and pixel size). Individual normalized values from all mice are presented in Supplemental Figure 1. The percent changes in Figure 5B represent the averaged values across three BIRI mice relative to three sham controls.

## Discussion

We successfully visualized and quantified lymphatic architecture in adult mouse kidneys by optimizing an antibody-based protocol. Three-dimensional volume rendering and image quantification revealed alterations in kidney lymphatic volume, diameter, length, and branching after ischemic kidney injury.

Previous investigations of lymphatic dynamics after ischemic kidney injury showed evidence of lymphangiogenesis through RNA and protein expression of lymphatic markers using standard immunofluorescence and 3D tissue cytometry.^6,7,22^ Three-dimensional tissue cytometry demonstrated that lymphangiogenesis occurs most prominently near the cortico-medullary junction, the site most susceptible to ischemic damage.^23^ Building on this work, we developed a method to elucidate the structural organization of the kidney lymphatic network after injury. Here, we show that lymphangiogenesis after acute ischemic injury results in increased LV volume, branching density, and total filament length. The increase in branch points and total filament length and decrease in average segment length presumably reflects *de novo* lymphangiogenesis. These new, shorter branches, as well as expansion of preexisting lymphatics, contribute to the increase in total lymphatic volume and may have functional implications for postinjury recovery.

Our method for imaging kidney LVs offers several advantages. Using antibodies provides flexibility and enables examination of various aspects of LVs without requiring reporter mice. Unlike other complex clearing protocols, iDISCO processing does not require monitoring of solution pH, hydrogel embedding, or electrophoresis.^21^ Moreover, processed samples remain shelf-stable in DBE for extended periods. Although the original iDISCO protocol requires a custom 3D-printed imaging chamber for confocal microscopy compatibility, we place tissues on a glass coverslip instead,^21^ increasing the working depth of field. For analysis, we selected *Imaris* for its user-friendly interface and powerful capabilities, although licensing costs may be prohibitive for some. Alternatively, matrix laboratory, VesselExpress,^24^ and ImageJ plugins offer feasible options for computational analysis (Table 1).

**Table 1:** Comparison of clearing methods: iDISCO versus proposed method

Feature	iDISCO (Renier *et al.*^21^ 2014)	Proposed Method	Advantages of Proposed Method
Sample preparation	Whole kidney	Quarter kidney	Shorter image acquisition times
Imaging chamber	3D-printed imaging chamber	Glass coverslip	Cost effective
Microscopy technique	Light sheet microscopy	Confocal microscopy	Shorter image acquisition times Reduced image file sizes
Analysis software	ImageJ, VAA3D, *Imaris*	NIS-Elements, *Imaris*	Limited computational expertise required
Quantification output	Individual cell quantification	Automated pipeline to detect diameter, volume, and branch points	Additional 3D measurements Reduced bias/time

3D, three-dimensional; iDISCO, immunolabeling-enabled three-dimensional imaging of solvent cleared organs; NIS-Elements, Nikon Imaging Software; VAA3D, 3D Visualization-Assisted Analysis.

By simultaneously visualizing two lymphatic markers, LYVE-1 and VEGFR-3, we demonstrate that the significant impact marker choice has on vessel quantification. Common lymphatic markers include Prox1, Podoplanin, LYVE-1, and VEGFR-3. Given our interest in vessel network structure, we focused on two membrane proteins, LYVE-1 and VEGFR-3. We omitted Podoplanin because of its significant nonlymphatic expression in kidney glomerular podocytes.^25^ This nonspecific expression, particularly when imaged at low resolution, can compromise accurate assessment of true lymphatic growth and lead to false positives in quantitative analyses.

The nuclear localization of Prox1 made it less suitable for quantification of multicellular vessel structures due to its discrete, punctate nuclear staining. The *Imaris* filament tracer requires contiguous features. In addition, higher resolution imaging is needed to distinguish each nucleus. This protocol minimizes acquisition times and prioritizes visualizing whole vessel structures rather than single LECs. Prox1 is also expressed in the loop of Henle in tubular epithelial cells, potentially causing nonspecific staining.^26^

This staining protocol can also incorporate markers to distinguish cortex from medulla, such as Megalin (low-density lipoprotein receptor-related protein 2) for proximal tubules (cortex), Nephrin for glomerular podocytes (cortex), and aquaporin 2 for collecting ducts (medulla).^27–29^ Either of these markers can be used to better distinguish between capillary lymphatics and hilar collecting ducts.

Using LYVE-1 and VEGFR-3 antibodies, quantified total vessel volume and filament length were consistently higher in injured kidneys regardless of the marker examined; however, changes in branch points and average segment length were notably more pronounced in LYVE-1^+^ LVs. Although both markers showed increased vessel volume and filament length, our data may suggest distinct mechanisms. The increase in VEGFR-3 volume likely reflects expansion of preexisting vessels, including VEGFR-3-expressing ascending vasa recta, which are described as “lymphatic-like.”^30^ By contrast, LYVE-1 structures may more accurately capture *de novo* lymphangiogenesis due to their increased branching density and decreased average segment length compared with sham controls. This interpretation is supported by the fact that ascending vasa rectas do not typically undergo the same *de novo* proliferative processes in response to injury as cortical lymphatics do. Interestingly, both markers in injured mice showed a decrease in average segment length compared with sham controls, indicating that newly formed lymphatic structures are shorter than already established LVs. Our findings indicate that changes in vessel branching and length, rather than diameter or volume, serve as more reliable indicators of *de novo* lymphatic formation. Therefore, we recommend LYVE-1 over VEGFR-3 when only a single lymphatic marker is used. For more comprehensive assessment of lymphatic dynamics, multiple lymphatic markers should be used.

Several considerations must be addressed when using this method. Good perfusion before tissue procurement is essential for high-quality optical clearing. Moreover, when the entire kidney cannot be used for whole-mount imaging, careful sectioning is critical to obtain a representative sample of the organ-wide lymphatic network. Standardizing tissue sectioning techniques and normalization to absolute tissue volumes can help minimize variability.

Our approach allows for visualization of lymphatic changes at a higher spatial resolution compared with traditional histologic methods. This enhanced resolution not only aids in anatomical studies, but also organ-wide visualization of lymphatic architecture which may offer important insights into lymphatic functional capacity, particularly in the absence of reproducible methods of assessing lymphatic drainage in small animal models. Additional studies on immune cell trafficking could serve as useful proxies for evaluating lymphatic function and provide mechanistic insights into how lymphatic remodeling affects kidney recovery after injury.

Despite its advantages, this method still has limitations. First, obtaining high-resolution images of large tissue volumes is challenging and time intensive. The larger free working distance of a 10× objective results in lower resolution images, which may omit smaller capillary lymphatics. Obtaining higher-resolution imaging of large volumes can be achieved by using light-sheet microscopes (*e.g*., Alpenglow Aurora) and higher magnification objectives. While resolution improves, imaging the same tissue size results in significantly longer scanning times and larger file sizes. Access to light-sheet microscopy can also be cost and time prohibitive. For studies requiring higher resolution, confocal microscopy with higher objectives (*e.g*., 40×) can capture focused areas of interest after initial low-magnification scans, providing high-resolution data acquisition while minimizing time and storage demands.

This protocol has been optimized to clear and image one quarter of a kidney. Choosing the appropriate immersion media to match RIs of cleared tissue is critical; if the correct medium is not chosen, spherical aberrations and distortions of important anatomic structures can occur.

Background fluorescence can affect thresholding accuracy when identifying lymphatic structures. Precise thresholding parameters must be established before filament tracing to ensure specificity and prevent false positives. In addition, uneven staining intensities between cortical and hilar lymphatic structures can occur, potentially leading to underrepresentation of certain vessel populations. Differential staining issues can easily be addressed through region-specific analysis in *Imaris* by isolating anatomical segments of interest in the surfaces tool.

Furthermore, precise sectioning and consistent positioning of kidney tissue for imaging is critical for consistent results. Observed variations in measurements may stem from differences in imaging depth, anatomical plane selection, and regional heterogeneity within the kidney. While our algorithm was optimized using an injured mouse model, which may introduce some variability when applied to other images, this approach ensures unbiased quantitative analysis across all samples.

Selection of LYVE-1 and VEGFR-3 enabled clear visualization of vessel structures but did not delineate individual LECs, as these are both membrane proteins. Prox-1, a nuclear transcription factor, could serve as a proxy for cell count if included in the staining panel. In addition, methanol dehydration inadequately preserves endogenous fluorescence; this affects techniques (*e.g*., lineage-tracing and fate mapping) that require fluorescence reporter mice. Endogenous signal can be amplified by staining with an antibody corresponding to the reporter (*i.e*., anti-red fluorescent protein or anti-green fluorescent protein).

We developed and optimized an antibody-based and semiautomated approach for visualizing kidney lymphatics using confocal microscopy. Although we used a BIRI model as proof of principle, this protocol can be applied to various disease models including AKI, CKD, lymphedema, and tumor models to investigate lymphatic changes across different pathologies.

## Supplementary Material

## Data Availability

All data are included in the manuscript and/or supporting information.

## Supplemental Material

Supplemental Figure 1. Individual mouse quantification of LV markers in sham (*n*=3) and BIRI (*n*=3) groups.

## References

[1] Lake BB Menon R Winfree S, .; KPMP Consortium. An atlas of healthy and injured cell states and niches in the human kidney. Nature. 2023;619(7970):585–594. doi:10.1038/s41586-023-05769-3 37468583 PMC10356613

[2] Marcinnò F Vergara C Giovannacci L Quarteroni A Prouse G. Computational fluid-structure interaction analysis of the end-to-side radio-cephalic arteriovenous fistula. Comput Methods Programs Biomed. 2024;249:108146. doi:10.1016/j.cmpb.2024.108146 38593514

[3] Polomska AK Proulx ST. Imaging technology of the lymphatic system. Adv Drug Deliv Rev. 2021;170:294–311. doi:10.1016/j.addr.2020.08.013 32891679

[4] Breslin JW Yang Y Scallan JP Sweat RS Adderley SP Murfee WL. Lymphatic vessel network structure and physiology. Compr Physiol. 2018;9(1):207–299. doi:10.1002/cphy.c180015 30549020 PMC6459625

[5] Russell PS Hong J Windsor JA Itkin M Phillips ARJ. Renal lymphatics: anatomy, physiology, and clinical implications. Front Physiol. 2019;10:251. doi:10.3389/fphys.2019.00251 30923503 PMC6426795

[6] Zarjou A Black LM Bolisetty S, . Dynamic signature of lymphangiogenesis during acute kidney injury and chronic kidney disease. Lab Invest. 2019;99(9):1376–1388. doi:10.1038/s41374-019-0259-0 31019289 PMC6716993

[7] Ghajar-Rahimi G Barwinska D Whipple GE, . Acute kidney injury results in long-term alterations of kidney lymphatics in mice. Am J Physiol Renal Physiol. 2024;327(5):F869–F884. doi:10.1152/ajprenal.00120.2024 39323387 PMC11563594

[8] Jacques SL. Optical properties of biological tissues: a review. Phys Med Biol. 2013;58(11):R37–R61. doi:10.1088/0031-9155/58/11/R37 23666068

[9] Chi J Crane A Wu Z Cohen P. Adipo-clear: a tissue clearing method for three-dimensional imaging of adipose tissue. J Vis Exp. 2018;137:58271. doi:10.3791/58271 30102289 PMC6126572

[10] Vélez-Fort M Rousseau CV Niedworok CJ, . The stimulus selectivity and connectivity of layer six principal cells reveals cortical microcircuits underlying visual processing. Neuron. 2014;84(1):238. doi:10.1016/j.neuron.2014.09.026 28898623 PMC5639145

[11] Ertürk A Becker K Jährling N, . Three-dimensional imaging of solvent-cleared organs using 3DISCO. Nat Protoc. 2012;7(11):1983–1995. doi:10.1038/nprot.2012.119 23060243

[12] Susaki EA Tainaka K Perrin D, . Whole-brain imaging with single-cell resolution using chemical cocktails and computational analysis. Cell. 2014;157(3):726–739. doi:10.1016/j.cell.2014.03.042 24746791

[13] Reinhold HS Hopewell JW van Rijsoort A. A revision of the spalteholz method for visualizing blood vessels. Int J Microcirc Clin Exp. 1983;2(1):47–52. PMID: 6381355 6381355

[14] Steinke H Wolff W. A modified spalteholz technique with preservation of the histology. Ann Anat. 2001;183(1):91–95. doi:10.1016/S0940-9602(01)80020-0 11206989

[15] Liu H Hiremath C Patterson Q, . Heterozygous mutation of Vegfr3 reduces renal lymphatics without renal dysfunction. J Am Soc Nephrol. 2021;32(12):3099–3113. doi:10.1681/ASN.2021010061 34551997 PMC8638391

[16] Jafree DJ Moulding D Kolatsi-Joannou M, . Spatiotemporal dynamics and heterogeneity of renal lymphatics in Mammalian development and cystic kidney disease. Elife. 2019;8:e48183. doi:10.7554/eLife.48183 31808745 PMC6948954

[17] Cheung MD Erman EN Moore KH, . Resident macrophage subpopulations occupy distinct microenvironments in the kidney. JCI Insight. 2022;7(20):e161078. doi:10.1172/jci.insight.161078 36066976 PMC9714795

[18] Melkonian AL Cheung MD Erman EN, . Single-cell RNA sequencing and spatial transcriptomics reveal unique subpopulations of infiltrating macrophages and dendritic cells following AKI. Am J Physiol Renal Physiol. 2025;328(6):F907–F920. doi:10.1152/ajprenal.00059.2025 40331777

[19] McCabe JT Desharnais RA Pfaff DW. Graphical and statistical approaches to data analysis for in situ hybridization. Methods Enzymol. 1989;168:822–848. doi:10.1016/0076-6879(89)68061-5 2725325

[20] Behrmann MS Trakselis MA. In vivo fluorescent TUNEL detection of single stranded DNA gaps and breaks induced by dnaB helicase mutants in Escherichia coli. Methods Enzymol. 2022;672:125–142. doi:10.1016/bs.mie.2022.02.021 35934472

[21] Renier N Wu Z Simon DJ Yang J Ariel P Tessier-Lavigne M. iDISCO: a simple, rapid method to immunolabel large tissue samples for volume imaging. Cell. 2014;159(4):896–910. doi:10.1016/j.cell.2014.10.010 25417164

[22] Black LM Winfree S Khochare SD, . Quantitative 3-dimensional imaging and tissue cytometry reveals lymphatic expansion in acute kidney injury. Lab Invest. 2021;101(9):1186–1196. doi:10.1038/s41374-021-00609-2 PMC8373805 34017058

[23] Venkatachalam MA Bernard DB Donohoe JF Levinsky NG. Ischemic damage and repair in the rat proximal tubule: differences among the S1, S2, and S3 segments. Kidney Int. 1978;14(1):31–49. doi:10.1038/ki.1978.87 682423

[24] Spangenberg P Hagemann N Squire A, . Rapid and fully automated blood vasculature analysis in 3D light-sheet image volumes of different organs. Cell Rep Methods. 2023;3(3):100436. doi:10.1016/j.crmeth.2023.100436 37056368 PMC10088239

[25] Tanaka K Tanaka M Watanabe N, . C-type lectin-like receptor (CLEC)-2, the ligand of podoplanin, induces morphological changes in podocytes. Sci Rep. 2022;12(1):22356. doi:10.1038/s41598-022-26456-9 36572741 PMC9792514

[26] Donnan MD Kenig-Kozlovsky Y Quaggin SE. The lymphatics in kidney health and disease. Nat Rev Nephrol. 2021;17(10):655–675. doi:10.1038/s41581-021-00438-y 34158633

[27] Sun J Hultenby K Axelsson J, . Proximal tubular expression patterns of megalin and cubilin in proteinuric nephropathies. Kidney Int Rep. 2017;2(4):721–732. doi:10.1016/j.ekir.2017.02.012 29142988 PMC5678615

[28] Ruotsalainen V Ljungberg P Wartiovaara J, . Nephrin is specifically located at the slit diaphragm of glomerular podocytes. Proc Natl Acad Sci U S A. 1999;96(14):7962–7967. doi:10.1073/pnas.96.14.7962 10393930 PMC22170

[29] Nielsen S Frøkiaer J Marples D Kwon TH Agre P Knepper MA. Aquaporins in the kidney: from molecules to medicine. Physiol Rev. 2002;82(1):205–244. doi:10.1152/physrev.00024.2001 11773613

[30] Kenig-Kozlovsky Y Scott RP Onay T, . Ascending vasa recta are Angiopoietin/Tie2-Dependent lymphatic-like vessels. J Am Soc Nephrol. 2018;29(4):1097–1107. doi:10.1681/ASN.2017090962 29237738 PMC5875961

