# Supplementary material for: Antibody-Based Imaging of Lymphatic Architecture in Murine Kidneys
Source: Kidney360. 2025 Aug 8;6(9):1586–95. doi: 10.34067/KID.0000000919 (PMC12483051; doi:10.34067/KID.0000000919)
Supplement: Supplementary file 1 [file kidney360-6-01586-s001.pdf]

Supplemental Figure 1

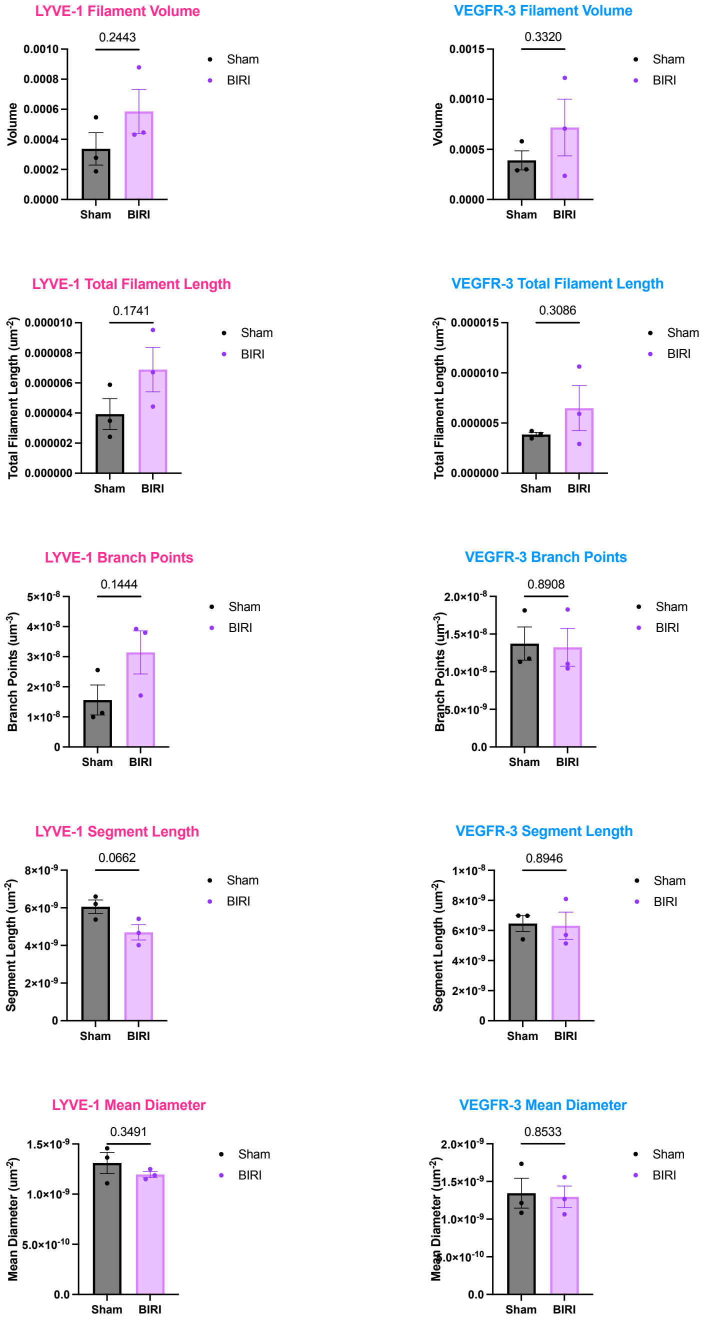

Supplemental Figure 1: Individual mouse quantification of lymphatic vessel markers in sham (n=3) and BIRI (n=3) groups. All raw values were divided by the absolute imaging volume; units represent normalized values. LYVE-1^{+} structures are shown in the left column and VEGFR-3^{+} structures in the right column. Parameters analyzed include total filament volume (sum), total filament length (sum), total number of branch points, average segment mean diameter, and average segment length. Statistical comparisons were conducted using unpaired t-tests in Prism version 9.4.0. Data represent mean ± standard error.
