# Supplementary material for: Antibody-Based Imaging of Lymphatic Architecture in Murine Kidneys
Source: Kidney360. 2025 Aug 8;6(9):1586–95. doi: 10.34067/KID.0000000919 (PMC12483051; doi:10.34067/KID.0000000919)
Supplement: Supplementary file 2 [file kidney360-6-01586-s002.pdf]

ASN American Society of Nephrology JASN Journal of the American Society of Nephrology KIDNEY360 Accessing Our World From Every Angle CJASN Clinical Journal of the American Society of Nephrology

ASN Journal Disclosure Form

As per ASN journal policy, I have disclosed any financial relationships or commitments I have held in the past 36 months as included below. I have listed my Current Employer below to indicate there is a relationship requiring disclosure. If no relationship exists, my Current Employer is not listed.

A. Agarwal reports the following:
Employer: University of Alabama at Birmingham; Consultancy: Dynamed - my role is to review content related to AKI for Dynamed and review updated materials prepared by the Dynamed editorial team for AKI topics.; I serve on the medical advisory boards of Creegh Pharmaceuticals, Alpha Young and Vexev.; Ownership Interest: Creegh Pharmaceuticals, Vexev; Research Funding: Genzyme/Sanofi Fabry Fellowship Award; Honoraria: Cornell, Mount Sinai, Univ of Virginia,; Patents or Royalties: I have a US patent that describes small molecule inducers of heme oxygenase-1 for the treatment of acute and chronic kidney disease.; Advisory or Leadership Role: Editorial Board of AJP Renal, Kidney Int and Lab Investigation.; I have served on the Advisory board of of Angion, Creegh Pharmaceuticals, Zydus, Bioporto, Vexev, Vera Therapeutics and Alpha Young, LLC.; and Other Interests or Relationships: My wife, Lisa Curtis was Past President for Women in Nephrology (2023-2024).

I understand that the information above will be published within the journal article, if accepted, and that failure to comply and/or to accurately and completely report the potential financial conflicts of interest could lead to the following: 1) Prior to publication, article rejection, or 2) Post-publication, sanctions ranging from, but not limited to, issuing a correction, reporting the inaccurate information to the authors' institution, banning authors from submitting work to ASN journals for varying lengths of time, and/or retraction of the published work.

Name: Anupam Agarwal
Manuscript ID: K360-2025-000162R2
Manuscript Title: Antibody-based Imaging of Lymphatic Architecture in Murine Kidneys
Date of Completion: June 4, 2025
Disclosure Updated Date: April 15, 2025

ASN
American Society of Nephrology
JASN
Journal of the American Society of Nephrology
KIDNEY360
Accessing Our World From Every Angle
CJASN
Clinical Journal of the American Society of Nephrology

ASN Journal Disclosure Form

As per ASN journal policy, I have disclosed any financial relationships or commitments I have held in the past 36 months as included below. I have listed my Current Employer below to indicate there is a relationship requiring disclosure. If no relationship exists, my Current Employer is not listed.

T. El-Achkar reports the following:
Employer: Indiana University; U.S Department of Veterans Affairs; Research Funding: NIH-NIDDK; VA Merit; Honoraria: Kyowa Kirin; Patents or Royalties: US Patent US 11,053,290 B2, : Modified tamm-horsfall protein and related compositions and methods of use ; Patent application (pending): Materials and methods for quantifying precursor Tamm-Horsfall Protein; Patent application (pending): Enhanced expression of alternative spliced uromodulin for therapeutic use;; and Other Interests or Relationships: American Physiological Society; American College of Physicians;.

I understand that the information above will be published within the journal article, if accepted, and that failure to comply and/or to accurately and completely report the potential financial conflicts of interest could lead to the following: 1) Prior to publication, article rejection, or 2) Post-publication, sanctions ranging from, but not limited to, issuing a correction, reporting the inaccurate information to the authors' institution, banning authors from submitting work to ASN journals for varying lengths of time, and/or retraction of the published work.

Name: Tarek M. El-Achkar
Manuscript ID: K360-2025-000162R2
Manuscript Title: Antibody-based Imaging of Lymphatic Architecture in Murine Kidneys
Date of Completion: July 8, 2025
Disclosure Updated Date: July 8, 2025

ASN American Society of Nephrology
JASN Journal of the American Society of Nephrology
KIDNEY360 Accessing Our World From Every Angle
CJASN Clinical Journal of the American Society of Nephrology

ASN Journal Disclosure Form

As per ASN journal policy, I have disclosed any financial relationships or commitments I have held in the past 36 months as included below. I have listed my Current Employer below to indicate there is a relationship requiring disclosure. If no relationship exists, my Current Employer is not listed.

J. George reports the following:
Employer: University of Alabama at Birmingham; Ownership Interest: Tesla; and Research Funding: Rapafusyn; Peel Therapeutics; Function Therapeutics; Young Therapeutics.

I understand that the information above will be published within the journal article, if accepted, and that failure to comply and/or to accurately and completely report the potential financial conflicts of interest could lead to the following: 1) Prior to publication, article rejection, or 2) Post-publication, sanctions ranging from, but not limited to, issuing a correction, reporting the inaccurate information to the authors' institution, banning authors from submitting work to ASN journals for varying lengths of time, and/or retraction of the published work.

Name: James F. George
Manuscript ID: K360-2025-000162R1
Manuscript Title: Antibody-based Imaging of Lymphatic Architecture in Murine Kidneys
Date of Completion: March 24, 2025
Disclosure Updated Date: June 26, 2024

ASN American Society of Nephrology
JASN Journal of the American Society of Nephrology
KIDNEY360 Accessing Our World From Every Angle
CJASN Clinical Journal of the American Society of Nephrology

ASN Journal Disclosure Form

As per ASN journal policy, I have disclosed any financial relationships or commitments I have held in the past 36 months as included below. I have listed my Current Employer below to indicate there is a relationship requiring disclosure. If no relationship exists, my Current Employer is not listed.

G. Ghajar-Rahimi reports the following:
Employer: University of Alabama at Birmingham

I understand that the information above will be published within the journal article, if accepted, and that failure to comply and/or to accurately and completely report the potential financial conflicts of interest could lead to the following: 1) Prior to publication, article rejection, or 2) Post-publication, sanctions ranging from, but not limited to, issuing a correction, reporting the inaccurate information to the authors' institution, banning authors from submitting work to ASN journals for varying lengths of time, and/or retraction of the published work.

Name: Gelare Ghajar-Rahimi
Manuscript ID: K360-2025-000162R1
Manuscript Title: Antibody-based Imaging of Lymphatic Architecture in Murine Kidneys
Date of Completion: March 24, 2025
Disclosure Updated Date: March 24, 2025

ASN
American Society of Nephrology
JASN
Journal of the American Society of Nephrology
KIDNEY360
Accessing Our World From Every Angle
CJASN
Clinical Journal of the American Society of Nephrology

ASN Journal Disclosure Form

As per ASN journal policy, I have disclosed any financial relationships or commitments I have held in the past 36 months as included below. I have listed my Current Employer below to indicate there is a relationship requiring disclosure. If no relationship exists, my Current Employer is not listed.

M. Kamocka reports the following:
Employer: IUPUI

I understand that the information above will be published within the journal article, if accepted, and that failure to comply and/or to accurately and completely report the potential financial conflicts of interest could lead to the following: 1) Prior to publication, article rejection, or 2) Post-publication, sanctions ranging from, but not limited to, issuing a correction, reporting the inaccurate information to the authors' institution, banning authors from submitting work to ASN journals for varying lengths of time, and/or retraction of the published work.

Name: Malgorzata Kamocka
Manuscript ID: K360-2025-000162R1
Manuscript Title: Antibody-based Imaging of Lymphatic Architecture in Murine Kidneys," submitted to the Kidney360
Date of Completion: March 24, 2025
Disclosure Updated Date: May 13, 2024

ASN
American Society of Nephrology
JASN
Journal of the American Society of Nephrology
KIDNEY360
Accessing Our World From Every Angle
CJASN
Clinical Journal of the American Society of Nephrology

ASN Journal Disclosure Form

As per ASN journal policy, I have disclosed any financial relationships or commitments I have held in the past 36 months as included below. I have listed my Current Employer below to indicate there is a relationship requiring disclosure. If no relationship exists, my Current Employer is not listed.

T. Lee reports the following:
Employer: University of Alabama at Birmingham and; Veterans Affairs Medical Center, Birmingham, AL; Consultancy: BD Bard Consultant; Boston Scientific; Xeltis; Venostent, and Humacyte; and Advisory or Leadership Role: Associate Editor, Kidney 360.

I understand that the information above will be published within the journal article, if accepted, and that failure to comply and/or to accurately and completely report the potential financial conflicts of interest could lead to the following: 1) Prior to publication, article rejection, or 2) Post-publication, sanctions ranging from, but not limited to, issuing a correction, reporting the inaccurate information to the authors' institution, banning authors from submitting work to ASN journals for varying lengths of time, and/or retraction of the published work.

Name: Timmy C. Lee
Manuscript ID: K360-2025-000162R1
Manuscript Title: Antibody-based Imaging of Lymphatic Architecture in Murine Kidneys
Date of Completion: March 24, 2025
Disclosure Updated Date: January 16, 2025

ASN
American Society of Nephrology
JASN
Journal of the American Society of Nephrology
KIDNEY360
Accessing Our World From Every Angle
CJASN
Clinical Journal of the American Society of Nephrology

ASN Journal Disclosure Form

As per ASN journal policy, I have disclosed any financial relationships or commitments I have held in the past 36 months as included below. I have listed my Current Employer below to indicate there is a relationship requiring disclosure. If no relationship exists, my Current Employer is not listed.

A. Melkonian has nothing to disclose.

I understand that the information above will be published within the journal article, if accepted, and that failure to comply and/or to accurately and completely report the potential financial conflicts of interest could lead to the following: 1) Prior to publication, article rejection, or 2) Post-publication, sanctions ranging from, but not limited to, issuing a correction, reporting the inaccurate information to the authors' institution, banning authors from submitting work to ASN journals for varying lengths of time, and/or retraction of the published work.

Name: Arin Melkonian
Manuscript ID: K360-2025-000162R1
Manuscript Title: Antibody-based Imaging of Lymphatic Architecture in Murine Kidneys
Date of Completion: March 24, 2025
Disclosure Updated Date: February 4, 2025
